# A type of magnetic electrochemical nano-biosensors for sensitive detection of heterogeneous nuclear ribonucleoprotein A1

**DOI:** 10.3389/fchem.2026.1879856

**Published:** 2026-07-22

**Authors:** Lei Sun, Zhixiang Lv, Zhou Wang, Li Wang, Zhan-ao Wu

**Affiliations:** 1 Danyang Maternal and Child Health Hospital, Zhenjiang, China; 2 The People’s Hospital of Danyang, Affiliated Danyang Hospital of Nantong University, Zhenjiang, China; 3 College of Vanadium and Titanium, Panzhihua University, Panzhihua, China; 4 Zhenjiang 359 Hospital, Zhenjiang, China

**Keywords:** aptamer, detection, electrochemical nano-biosensor, Fe_3_O_4_/α-Fe_2_O_3_ magnetic heterogeneous nanorods, hnRNP A1

## Abstract

**Introduction:**

The detection of tumor markers has become an important non-invasive detection method for early detection, diagnosis, and prognosis and treatment of tumors.

**Methods:**

In this work, we designed and developed a novel type of electrochemical nano-biosensors characterized by magnetically driven self-assembly based on Fe_3_O_4_/α-Fe_2_O_3_ (ferriferrous oxide/α-iron trioxide, FOαIT) magnetic heterogeneous nanorods fabricated with CTAB as a restricted growth agent for sensitive detection of heterogeneous nuclear ribonucleoprotein A1 (hnRNP A1). Firstly, FOαIT@Au magnetic nanocomposites (MNCs) were prepared via the hydrothermal and calcination-reduction process with Au nanoparticles (AuNPs) modified on the surface. With the help of Au-S bonding, ssDNA/BC15 was immobilized to form FOαIT@Au/ssDNA/BC15. To avoid the effect of nonspecific sites to the detection, BSA was employed to block them and form FOαIT@Au/ssDNA/BC15/BSA nano-biosensors. Utilizing the stronger linkage force of hnRNP A1 with BC15 than that of FOαIT@Au/ssDNA, the sensitive and accurate detection of hnRNP A1 was realized.

**Results and Discussion:**

The nano-biosensors revealed a decent linear relationship with a lower LOD of 0.103 pg/mL, higher sensitivity, excellent selectivity, satisfactory repeatability (RSD ≤1.55%), dependable long-term stability, and the accurate detection of spiked samples, revealing the promising application prospect in the clinical examination.

## Graphical Abstract

**Figure:**
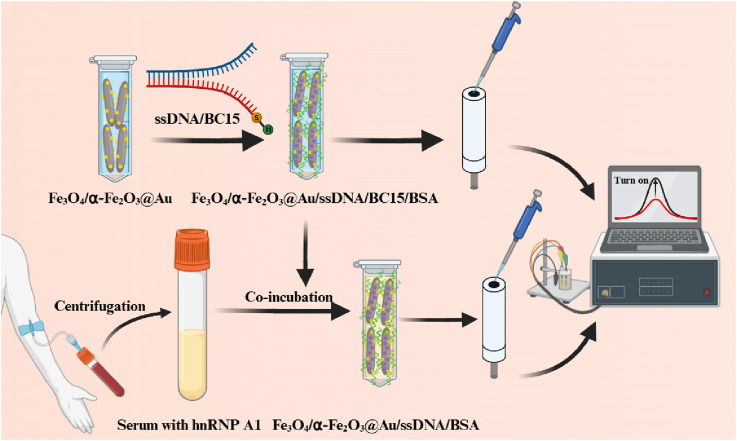
Fe_3_O_4_/α-Fe_2_O_3_ MHNRs were prepared and the aptamer was combined on their surface by Au-S bond to form magnetic nano-biosensors for hnRNP A1 detection, and the constructed nano-biosensors exhibited lower LOD, excellent specificity, stability, reproducibility, and satisfactory recovery rate in human serum samples, suggesting the promising application prospect in clinical examination.

## Highlights

- A magnetic electrochemical nano-biosensor was developed for rapid detection of hnRNP A1.
- Fe_3_O_4_/α-Fe_2_O_3_ MHNRs were prepared to amplify signal for electrochemical detection.
- The nano-biosensors demonstrated excellent detection properties.

## 1 Introduction

Cancer has always been one of the major causes of mortality worldwide. According to the estimation of GLOBOCAN 2022, nearly 20 million cancer cases were diagnosed worldwide annually, and nearly 800,000 died from cancer (El-Wakil et al., 2025; Martinez et al., 2026). Cancer is always a threat to human health and life. Therefore, the prevention and prognostic treatment of cancers increasingly reveal their great importance. Many approaches have been developed for the diagnosis of cancers. Among them, the electrochemical method displays significant (Chen et al., 2026; Noreen et al., 2025). In the electrochemical analysis process, the detection of specific cancer biomarkers has attracted wide attention.

The heterogeneous nuclear ribonucleoprotein A1 (hnRNP A1) plays a vital role in the hnRNPs family. Its functional domains play roles in transcription, splicing, and cytoplasmic transport in a combinatorial manner (Ryu et al., 2021; Yatsu et al., 2025). It can mutually affect the noncoding RNA and participate in malignant proliferation of various cancer cells and neuropathy (Siculella et al., 2023), including gastric cancer (Qiu et al., 2013), colorectal cancer (Lee et al., 2020), hepatocellular carcinoma (Cao et al., 2023; Ke et al., 2019), lung cancer, multiple myeloma, leukemia (Ji et al., 2019), etc. Hence, hnRNP A1 became an effective cancer biomarker and therapeutic target for the prevention and prognostic treatment of cancers (Lu et al., 2022; Ma et al., 2009).

With the development of nanoscience and nanotechnology, nanomaterials have been applied in biosensor structures to promote the rapid movement of electrons from the active reaction center to the surface of the electrode, thereby improving the stability and sensitivity (Zhao et al., 2025). In particular, the magnetic nanomaterials can not only realize magnetically induced self-assembly onto the magnetic glass carbon electrode (MGCE), but also increase the electroconductivity of the electrochemical reaction, thereby further enhancing the sensitivity of electrochemical detection (Zhang et al., 2025).

**SCHEME 1:**
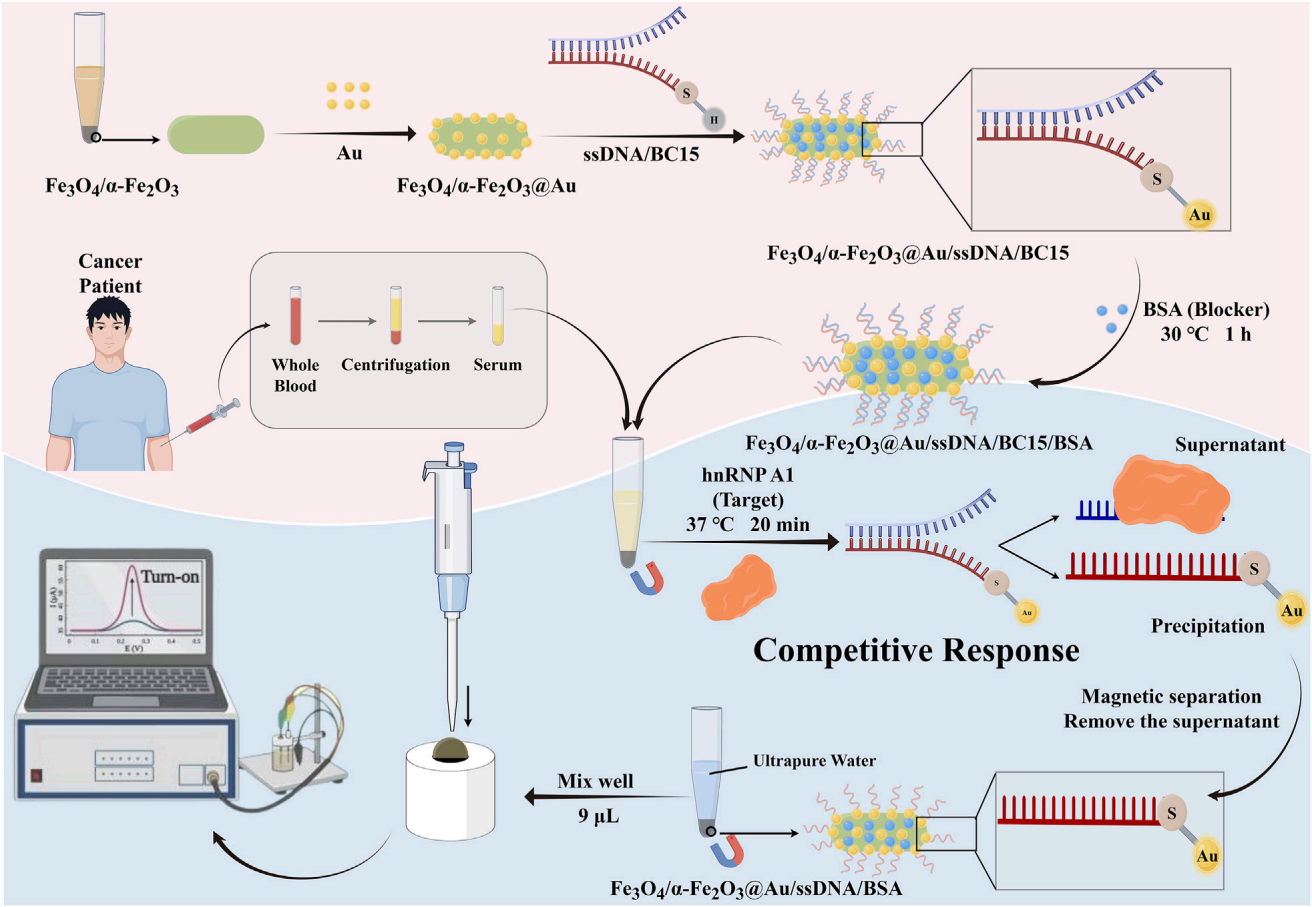
Construction and detection process of FOαIT@Au/ssDNA/BC15/BSA nano-biosensors for hnRNP A1.

In this work, we fabricated Fe_3_O_4_/α-Fe_2_O_3_ (ferriferrous oxide/α-iron trioxide, FOαIT) magnetic heterogeneous nanorods (MHNRs) with CTAB as restricted growth agent. Based on FOαIT MHNRs, FOαIT@Au magnetic nanocomposites (MNCs) were produced via the reduction of HAuCl_4_ by sodium borohydride. With the help of Au-S binding, the novel designed aptamers of ssDNA/BC15 possessing sulfydryl groups were immobilized onto the surfaces of FOαIT@Au MNCs to form FOαIT@Au/ssDNA/BC15 MNCs. Subsequently, bovine serum albumin (BSA) was applied to block non-specific sites, and the FOαIT@Au/ssDNA/BC15/BSA nano-biosensors were successfully constructed. Finally, the nano-biosensors were incubated with hnRNP A1, hnRNP A1 competitively combined with BC15, and BC15 fell off from the nano-biosensors to form FOαIT@Au/ssDNA/BSA MNCs. The current changes of FOαIT@Au/ssDNA/BC15/BSA and FOαIT@Au/ssDNA/BSA reflected the amount of hnRNP A1, and a “turn-on” type detection of hnRNP A1 was realized. The construction and detection process of the nano-biosensors for hnRNP A1 were shown in Scheme 1.

## 2 Experimental

### 2.1 Fabrication and characterization of FOαIT@Au MNCs

FOαIT@Au MNCs were prepared via hydrothermal treatment and subsequent calcination–reduction, according to the previous works (Wang et al., 2025b; Zhang et al., 2026). Typically, 4 mmol of FeCl_3_.6H_2_O and 7.5 mmol of cetyltrimethylammonium bromide (CTAB) were completely dissolved in double-distilled water (DDW) of 70 mL. The obtained solution was transferred into a hydrothermal reactor and hydrothermally reacted for 2 h at 120 °C. After the reaction was completed, a suspension was formed. The suspension was separated, the solid was alternately washed with alcohol and DDW, dried and ground. Then β-FeOOH nanorods (NRs) were obtained. Subsequently, β-FeOOH NRs were uniformly mixed with maltose based on their mass ratio of 1:10, followed by calcination at 400 °C for 2 h. The product was ground, and FOαIT MHNRs were obtained.

1 g polyethyleneimine (PEI) was dissolved in 100 mL DDW, and 50 mg FOαIT MHNRs were added to the as-prepared solution and treated by ultrasonication for 30 min. The suspension was hydrothermally reacted for 1 h at 90 °C. After that, the suspension was magnetically separated and washed three times with DDW, the solid was dried and ground, and the intermediates of FOαIT@PEI MNCs were obtained. 15 mg FOαIT@PEI MNCs were dispersed into 150 mL DDW, ultrasonically dispersed for 10 min in the ice bath, then 1 mL HAuCl_4_ solution was added and the mixture was further ultrasonically dispersed for 30 min. Finally, 9 mL NaBH_4_ solution (0.075%) was slowly added dropwise and mechanically stirred until the black mixture was formed. The suspension was magnetically separated, the solid was dried and ground, and FOαIT@Au MNCs were obtained.

The morphologies and composition analyses of FOαIT MHNRs and FOαIT@Au MNCs were investigated with scanning electron microscopy (SEM) and transmission electron microscopy (TEM), their phase identifications were characterized by X-ray diffraction (XRD), and their magnetic properties were measured by vibrating sample magnetometer (VSM).

### 2.2 Construction of electrochemical nano-biosensor for hnRNP A1 detection

SsDNA (5′-SH-(CH_2_)_6_-TTTTTTTTTACATCCCGCCTGCCTGGCCACA-3′) and BC15–31 (BC15: 5′-TGT​GGC​GAG​GTA​GGT​GGG​GTG​TGT​GTG​TAT​C-3′) were separately dissolved in TE buffer to form ssDNA and BC15 solutions, both at a concentration of 40 μM. A 10 mM tris(2-carboxyethyl)phosphine (TCEP) solution in DDW was added dropwise to the ssDNA solution at a volume ratio of TCEP to ssDNA of 1:100 to guarantee the presence of sulfhydryl groups for immobilization via Au–S binding. Subsequently, the equal volumes of the ssDNA solution and BC15 solution were mixed and incubated for 5 min at 95 °C, naturally cooled to room temperature (the annealing rate of about 35 ^°^C/h), and the double-stranded ssDNA/BC15 aptamers assembled sulfydryl were generated. 15 μL ssDNA/BC15 aptamer solution was incubated with 15 μL FOαIT@Au suspension (20 mg/mL) at 4 °C for 12 h to form FOαIT@Au/ssDNA/BC15 MNCs. Magnetic separation was conducted to remove the supernatant. The product was rinsed with PBS to remove the uncombined ssDNA/BC15 aptamers, FOαIT@Au/ssDNA/BC15 MNCs were dispersed in DDW, and 30 μL of BSA solution in PBS (0.25 wt%) was introduced and incubated at 30 °C for 1 h. In the same way, the suspension was magnetically separated, and the supernatant was removed. Then, the same washing process was repeated to remove the uncombined BSA, and the FOαIT@Au/ssDNA/BC15/BSA nanoprobes were obtained. The nanoprobes were added to 30 μL of hnRNP A1 solution in TE buffer and incubated at 37 °C for 1 h. BC15 combined with hnRNP A1 (BC15-hnRNP A1) and fell off from the nanoprobes, repeated the previous operations, and the uncombined hnRNP A1 and BC15-hnRNP A1were washed with PBS. At last, FOαIT@Au/ssDNA/BSA was obtained. Each step for MGCE, FOαIT, FOαIT@Au, FOαIT@Au/ssDNA/BC15/BSA, and FOαIT@Au/ssDNA/BSA was detected by CHI760 E electrochemical workstation with MGCE, Pt, and Ag/AgCl as work, auxiliary, and reference electrodes. Every substance was dispersed in 30 μL DDW, and 9 μL suspension was dropped onto MGCE with the cross-sectional diameter of 3 mm to detect the current three times. Therein, the electrolyte contained 5 mM K_4_Fe(CN)_6_·3H_2_O, 5 mM K_3_Fe(CN)_6_, and 0.1 M KCl, the scan voltage of cyclic voltammetry (CV) was −0.1 V–0.7 V with a scan rate of 100 mV/s. The frequency range of electrochemical impedance spectroscopy (EIS) was 0.1 Hz–10 kHz with a signal amplitude of 5 mV, the voltage of differential pulse voltammetry (DPV) analysis was set from initial potential of 0 V to final potential of 0.6 V with incremental potential of 0.004 V, pulse amplitude of 0.05 V, pulse width of 0.06 s, sample width of 0.02 s, pulse period of 0.5 s, quiet time of 2 s, and sensitivity of 10^−4^ A/V (Xu et al., 2024).

## 3 Results and discussion

### 3.1 Characteristics of β-FeOOH NRs and Fe_3_O_4_/α-Fe_2_O_3_@Au MNCs

**FIGURE 1:**
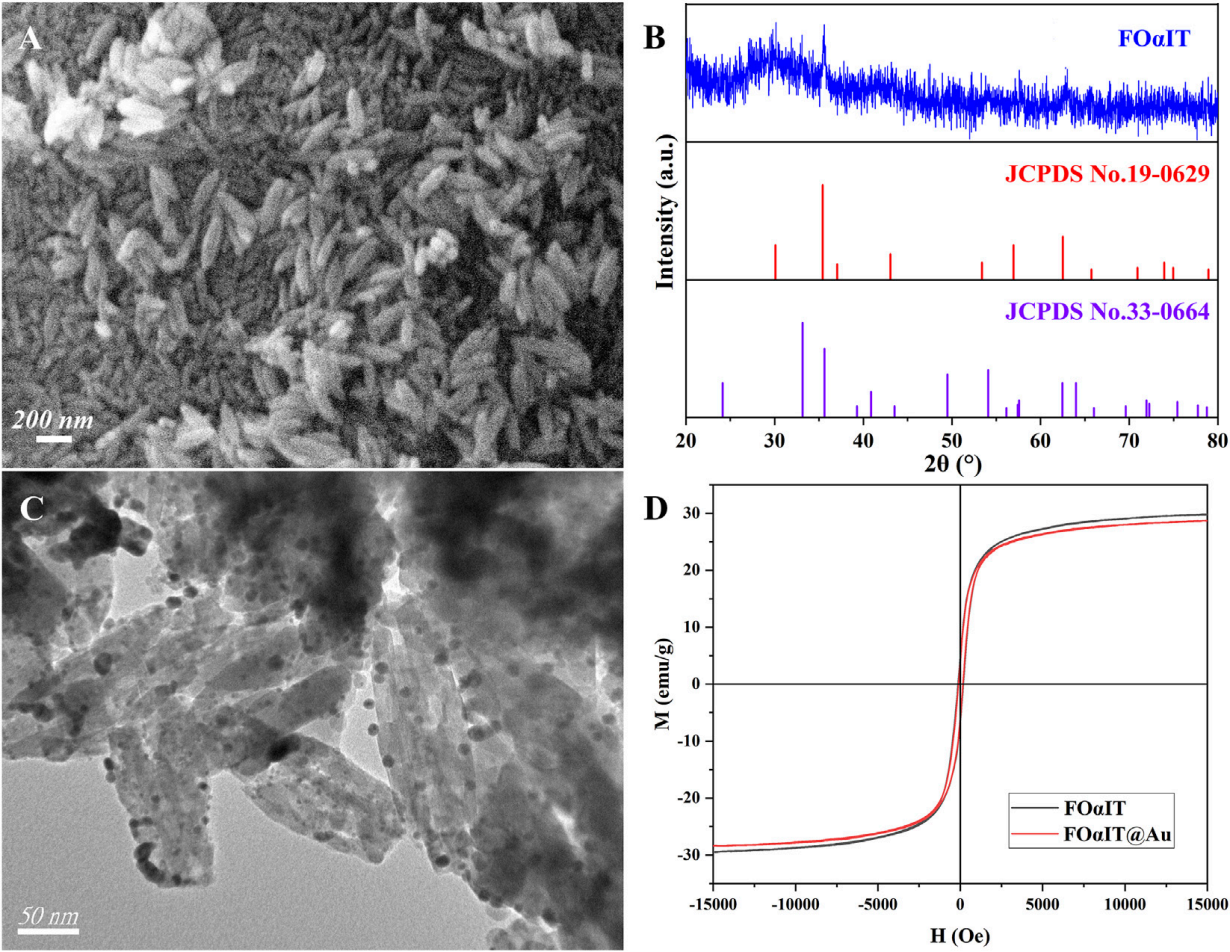
SEM morphology **(A)** of β-FeOOH NRs, XRD patterns **(B)** of FOαIT MHNRs, TEM image **(C)** of FOαIT@Au MNCs, and the hysteresis loops **(D)** of FOαIT MHNRs and FOαIT@Au MNCs.

The characteristics of β-FeOOH NRs and FOαIT@Au MNCs were displayed in Figure 1. The SEM morphology of β-FeOOH nanomaterials was shown in Figure 1A. Obviously, the β-FeOOH nanomaterials revealed a rod shape with an average diameter and length of 48 nm and 171 nm. Their rod structure provided the models for the FOαIT MHNRs. The XRD pattern of FOαIT MHNRs was presented in Figure 1B. The XRD diffraction peaks of FOαIT MHNRs at 24.1 °, 33.2 °, 35.6 °, 40.9 °, 49.5 °, 54.1 °, 57.6 °, 62.4 °, 64.0 °, 71.9 °, and 75.4 ° were corresponding to α-Fe_2_O_3_ reference PDF card (JCPDS No.33–0,664), which proved the presence of α-Fe_2_O_3_ in the MHNRs. However, the α-Fe_2_O_3_ reference PDF card showed that the diffraction intensity ratio at 33.2 ° and 35.6 ° was 10:7, while, the diffraction intensity ratio at 33.2 ° and 35.6 ° of FOαIT MHNRs was far less than 10:7 (Sun et al., 2026), the reason for this was that the XRD diffraction peak of Fe_3_O_4_ at 35.6 ° greatly enhanced the diffraction intensity at 35.6 °, at the same time, The XRD diffraction peaks of FOαIT MHNRs at 30.1 ° and 43.1 ° obviously corresponded to Fe_3_O_4_ reference PDF card (JCPDS No.19-0629), which revealed that the present of Fe_3_O_4_ in the MHNRs. And all the results also certified the successful fabrication of FOαIT MHNRs. The TEM image of FOαIT@Au MNCs was shown in Figure 1C, obviously, the FOαIT MHNRs retained the rod-like structure, which accorded with the conclusion from the analysis of SEM morphology. And the blank points on the surfaces of FOαIT MHNRs were Au nanoparticles (AuNPs) with an average size of 5 nm, which proved the formation of FOαIT MHNRs. The hysteresis loops of FOαIT MHNRs and FOαIT@Au MNCs were revealed in Figure 1D. The saturation magnetization (Ms) of FOαIT MHNRs reached 29.8 emu/g, and that of FOαIT@Au MNCs attained 28.7 emu/g, reducing 1.1 emu/g, which was due to the compensation of lattice imperfection by AuNPs, and this also revealed the loading of AuNPs onto the surfaces of FOαIT MHNRs.

**FIGURE 2:**
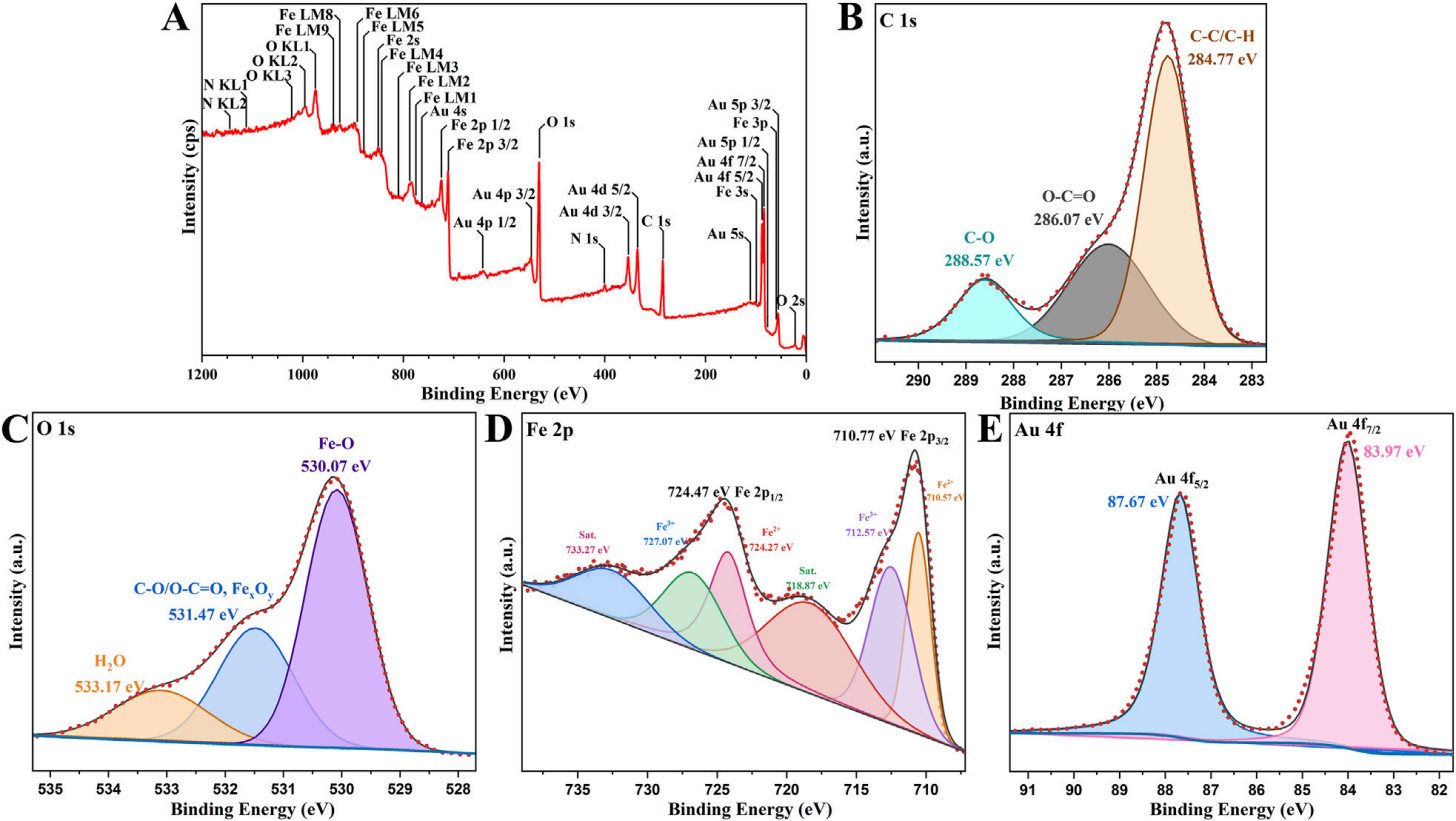
XPS spectra of α-Fe_2_O_3_/Fe_3_O_4_@Au nanocomposites: survey scan **(A)**, C 1s **(B)**, O 1s **(C)**, Fe 2p **(D)**, and Au 4f **(E)**.

To validate the successful preparation of FOαIT@Au MNCs, XPS was employed to investigate the elemental and phase composition of product. The full spectrum scan of XPS was shown in Figure 2A, all the characteristic peaks for Fe, O, and Au elements were exhibited and calibrated according to the C 1s peak with a binding energy of 284.77 eV. And the spectra of C 1s, O 1s, Fe 2p, and Au 4f were analyzed, fitted and calibrated. Figure 2B revealed C 1s splitting peaks corresponded to C-C/C-H, C-O and O-C=O, while Figure 2C displayed O 1s splitting peaks corresponded to Fe-O, C-O/C-O=C, and H_2_O. Figure 2D revealed the peaks observed at the binding energy of 710.77 eV and 724.47 eV, which corresponded to Fe 2p_3/2_ and Fe 2p_1/2_, respectively. Among them, the splitting peaks at 710.57 eV and 724.27 eV were attributed to Fe(II), while those at 712.57 eV and 727.07 eV belonged to Fe(III), respectively, along with two satellite peaks at 718.87 and 733.27 eV. These results suggested the existences of α-Fe_2_O_3_ and Fe_3_O_4_ phases in the product, i.e., indicating the formation of FOαIT MHNRs. What’s more, the deconvolution curve of the Au 4f in Figure 2E exhibiting the peaks at 83.97 eV and 87.67 eV were assigned to Au 4f_7/2_ and Au 4f_5/2_, respectively; which demonstrated the successful preparation of FOαIT@Au MNCs.

### 3.2 Electrokinetics

**FIGURE 3:**
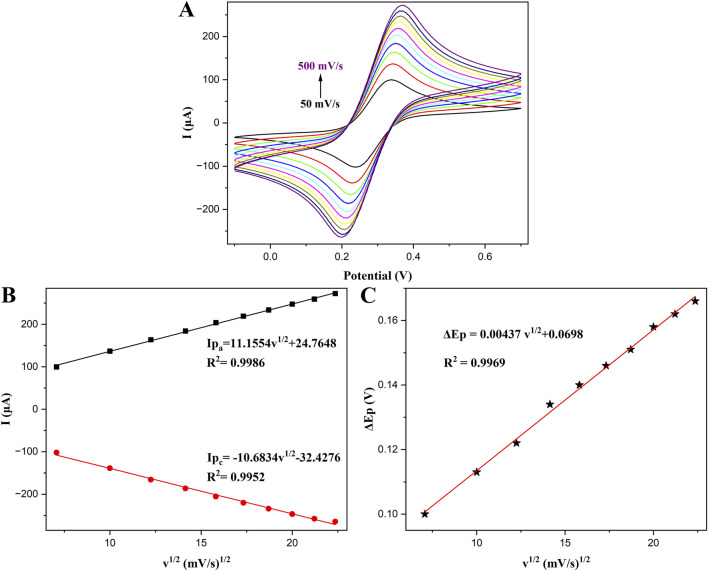
CV analysis **(A)** of bare MGCE with different scan rates, and the corresponding Ipa and Ipc peak current curves **(B)** and the changes (ΔEp) in peak potential **(C)** with the square roots of scan rates.

To profoundly explore the electron transfer kinetics for the electrochemical detections, CV curves for the bare MGCE were examined with various scan rates (*ν*) of 50 mV/s–500 mV/s. As demonstrated in Figure 3A, the anodic peak currents (*Ip*_*a*_) and the cathodic peak currents (*Ip*_*c*_) of the bare MGCE exhibited excellent linear relationships (*R* ^2^ = 0.9986 and 0.9952, respectively), suggesting that the electrolysis was a typical diffusion-controlled redox process. The electrolytic area of MGCE remained constant, and the diffusion coefficient (*D*) could be calculated according to the Randles Sevcik equation as Equation 1 (Wang et al., 2025a).

$$Ip=2.69\times 10^{5}\cdot n^{3/2}\cdot A\cdot D^{1/2}\cdot C\cdot \nu^{1/2}$$ (1)

wherein *C* was the molar concentration of the redox active substance (0.005 M), *A* represented the effective area of the electrode (0.0707 cm^2^), *n* was the electron transfer number (n = 1), *D* was the diffusion coefficient, and v was the scan rate (Yue et al., 2025).

The linear fitting curves (Figure 3B) of Ip_a_ and Ip_c_ vs. v^1/2^ for the bare MGCE describing slopes and intercepts were given by and Equation 3.

$${\text{Ip}}_{a}=11.1554\cdot v^{1/2}+24.7648\;\left(R^{2}=0.9986\right)$$ (2)

$${\text{Ip}}_{c}=‐10.6834\cdot v^{1/2}\;‐\;32.4276\;\left(R^{2}=0.9952\right)$$ (3)

Compared Equation 1 with Equations 2, 3, the average D was calculated as 0.013 cm^2^/s, which indicated the larger charge transfer ability of the electrolyte.

While, the relationship of the difference value ΔEp (ΔEp = Epa–Epc) for redox peaks’ potentials (Epa and Epc) vs. the square root of the scan rate was depicted in Figure 3C, the ΔEp curve revealed excellent linearity as Equation 4, suggesting a simple and easy transfer of electrons between the electrolyte and the surface of electrodes (Saidi et al., 2025). And, the values of ΔEp were no less than 0.10 V, and ΔEp increased with the increase of scan rate, revealing an inhibited electron transfer at the interface of MGCE (Turunc et al., 2026).

$$\Delta \text{Ep}=0.00437\cdot v^{1/2}+0.0698\;\left(R^{2}=0.9969\right)$$ (4)

### 3.3 Feasibility of the nano-biosensors

**FIGURE 4:**
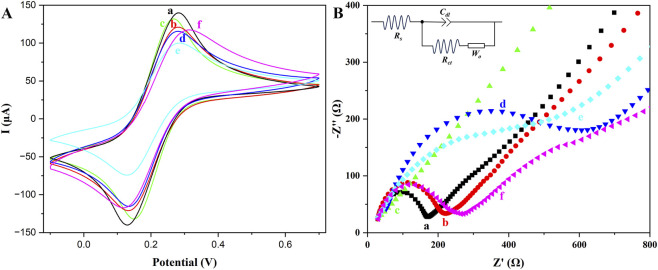
CV **(A)** and Nyquist plots **(B)** of bare MGCE (a), MGCE/FOαIT (b), MGCE/FOαIT@Au (c), MGCE/FOαIT@Au/ssDNA/BC15 (d), MGCE/FOαIT@Au/ssDNA/BC15/BSA (e), and MGCE/FOαIT@Au/ssDNA/BSA (f).

The CV and EIS analyses of all the substances at each construction step were investigated as shown in Figure 4. In the CV image (Figure 4A), the current peak of MGCE/FOαIT (curve b) was smaller than that of bare MGCE (curve a), the loading of FOαIT MHNRs onto MGCE obviously hindered the diffusion of [Fe(CN)_6_]^3-/4-^ from electrolyte to the surface of MGCE due to steric hindrance. The current peak of MGCE/FOαIT@Au (curve c) was tremendously increased compared with that of MGCE/FOαIT, revealing the outstanding conductivity of Au. The current peak of MGCE/FOαIT@Au/ssDNA/BC15 (curve d) was decreased owing to the loading of ssDNA/BC15. When BSA was employed to block the nonspecific sites, the current peak (curve e) was decreased, which suggested that the nonspecific sites were present. When hnRNP A1 competitively reacted with BC15, BC15 fell off from FOαIT@Au/ssDNA/BC15/BSA to form FOαIT@Au/ssDNA/BSA, so their current peak (curve f) was drastically enhanced. The Nyquist plots for various electrodes were displayed in Figure 4B, and the equivalent circuit diagram was inserted into inset of Figure 4B. The diameter of the semicircle was positively connected with the resistance of charge transfer. The smaller the diameter was, the smaller the resistance was; the larger the diameter was, the larger the resistance was. The charge-transfer resistances (R_ct_) were simulated and calculated, the R_ct_ for the bare MGCE was 87.4 Ω, and R_ct_ for MGCE/FOαIT reached 191.8 Ω, which revealed the successful self-assembling of FOαIT on the surface of MGCE. Subsequently, AuNPs loading led to the R_ct_ (135.1 Ω) increase of MGCE/FOαIT@Au. With the stepwise loadings of BC15 and BSA, R_ct_ reached 219.7 Ω and 268.5 Ω; and when hnRNP A1 was present, the R_ct_ value decreased to 208.9 Ω, which decreased 29.6 Ω compared with that of MGCE/FOαIT@Au/ssDNA/BC15/BSA. The change process was fully consistent with the variation trend of the CV current signal. Especially in the last step, the presence of hnRNP A1 as the detection object generated a large change in the current signal, which provided the possibility of detection.

### 3.4 Optimization of key factors for the construction of nano-biosensors

**FIGURE 5:**
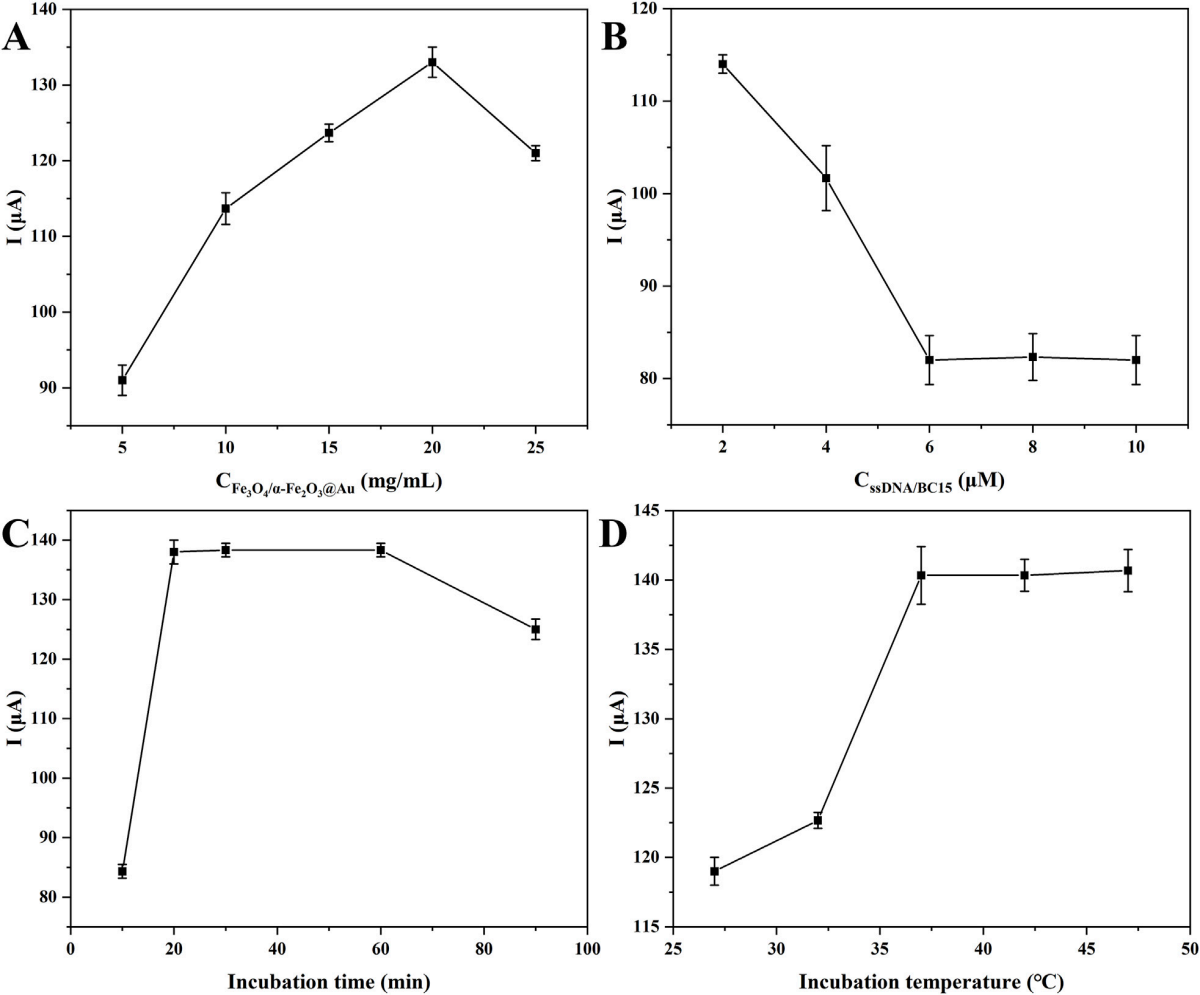
Optimization of FOαIT@Au concentration **(A)**, ssDNA/BC15 concentration **(B)**, the incubation time **(C)** and incubation temperature **(D)** of FOαIT@Au/ssDNA/BC15/BSA and hnRNP A1.

For more accurate detection, the construction factors for MGCE/FOαIT@Au/ssDNA/BC15/BSA, including the concentrations of FOαIT@Au and ssDNA/BC15, as well as incubation conditions (temperature and time) of hnRNP A1 were optimized as shown in Figure 5. Figure 5A showed the change in the current peak signal for the detection of FOαIT@Au. With the increasing concentration of FOαIT@Au suspension, the current signal increased, and achieved at 20 mg/mL. The reason for the increase was that the increase of FOαIT@Au resulted in more AuNPs, and Au was a better conductive material, therefore the increase of the current signal was inevitable. However, with the continuous increase of FOαIT@Au, the current signal decreased, which was owing to the saturation of FOαIT@Au on the surface of MGCE, more FOαIT@Au could not arrive the surface, on the contrary, FOαIT@Au formed multilayers and adhered to the surface of MGCE, after all the steric hindrance of multilayer FOαIT@Au was larger, which prevented the migration of [Fe(CN)_6_]^3-/4-^ from electrolyte to MGCE. Therefore, 20 mg/mL of FOαIT@Au suspension was employed for the detection of hnRNP A1. The concentration of ssDNA/BC15 was optimized as shown in Figure 5B, the current signal decreased with the increase of the concentration, and remained almost immovability when the concentration was equal to or greater than 6 μM, which suggested that more ssDNA/BC15 could not further improve the current signal as the concentration was greater than 6 μM. Therefore, 6 μM of ssDNA/BC15 concentration was employed as the optimal condition for the construction of the nano-biosensor.

The incubation time and the incubation temperature of FOαIT@Au/ssDNA/BC15/BSA and hnRNP A1 were optimized as shown in Figures 5C,D, respectively. At the initial stage, with the prolongation of incubation time, the combination of FOαIT@Au/ssDNA/BC15/BSA and hnRNP A1 increased, and the combination was completed within 20 min. When the incubation time exceeded 60 min, the related protein and nucleic acid might be inactivated, the inactivated substances reduced the electroconductivity, and then the current signal reduced. While, the increase of incubation temperature benefited the combination of FOαIT@Au/ssDNA/BC15/BSA and hnRNP A1, and as the incubation temperature reached 37 °C, their combination obtained an excellent state, the higher temperature was unnecessary. So, the incubation time and incubation temperature were valued as 20 min and 37 °C.

### 3.5 Detection performance of nano-biosensor for hnRNP A1

**FIGURE 6:**
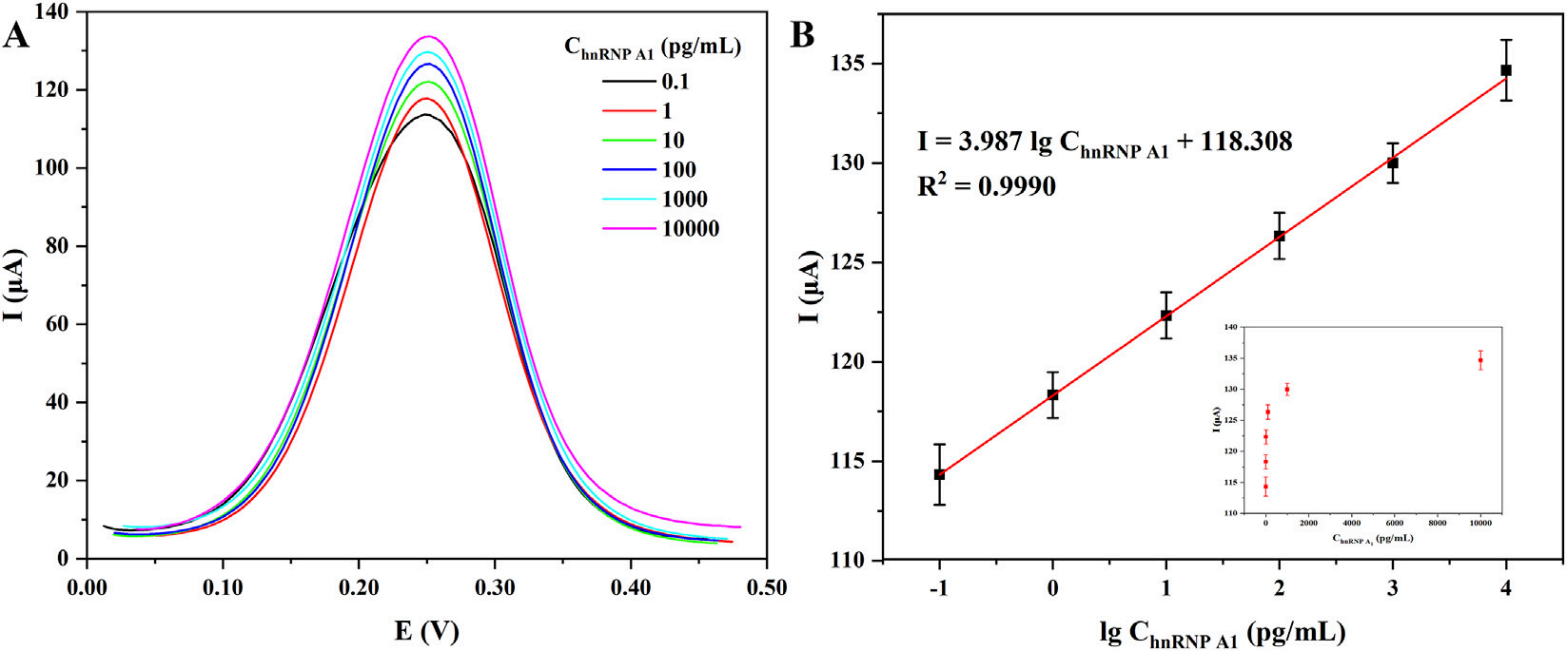
DPV curves **(A)** and the linear relationship **(B)** of nano-biosensor for various concentrations of hnRNP A1.

Under the optimized construction conditions, the FOαIT@Au/ssDNA/BC15/BSA nano-biosensors detected various concentrations (0.1 pg/mL, 1 pg/mL, 10 pg/mL, 100 pg/mL, 1 ng/mL, and 10 ng/mL) of hnRNP A1 by the DPV technique. The current signals for all the concentrations were revealed in Figure 6A. Obviously, the current signal gradually increased with the increase in hnRNP A1 concentration. The linear relationship between the current and the logarithmic value of hnRNP A1 concentration was shown in Figure 6B, which revealed a decent linear relationship with the fitting variance (R^2^) of 0.9990 in the range of 0.1 pg/mL–10 ng/mL, and the linear equation was I (μA) = 3.897 l g C_hnRNP A1_ (pg/mL) + 118.308. The limit of detection (LOD) and the limit of quantitation (LOQ) were calculated as 0.103 pg/mL and 0.344 pg/mL based on LOD = 3σ/slope and LOQ = 10σ/slope (slope referred to that of linear equation, while σ was the standard deviation of the intercept for the linear equation), respectively, revealing the lower LOD and LOQ of the nano-biosensors for hnRNP A1 detection, suggesting higher sensitivity of the detection. The detection performances of the related assays for hnRNP A1 were listed in Table 1. The hnRNP A1 nano-biosensors presented a lower detection range compared with the other two methods, which proved that the detection of hnRNP A1 could be carried out under a lower concentration, suggesting that the nano-biosensors could be applied in the early diagnosis in clinical examination. All these results demonstrated the promising prospects of the nano-biosensors for the prevention and treatment of cancers.

**TABLE 1:** Comparison of the present work with the other assays for hnRNP A1 detection.

Analysis component	Methods	Linear range	LOD	References
Apt/hnRNP A1/Anti-hnRNP A1	SPR	0.25 nM–5 nM	0.22 nM	Lee et al. (2020)
LoC-SERS + Apt-hnRNP A1	SERS	1 pg/mL–1 μg/mL	0.031 pg/mL	Cao et al. (2024)
α-Fe_2_O_3_/Fe_3_O_4_@Au + aptamer	DPV	0.1 pg/ml–10 ng/mL	0.103 pg/mL	This work

### 3.6 Selectivity, repeatability, stability, and the detection of spiked samples

**FIGURE 7:**
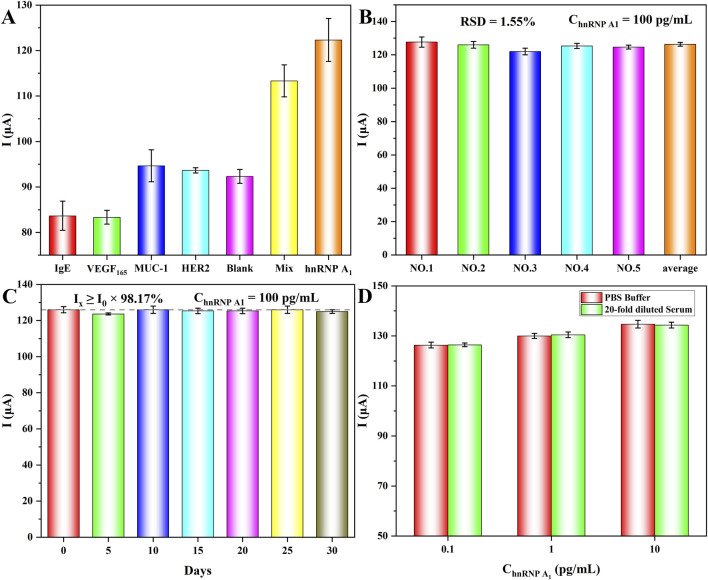
Selectivity **(A)**, stability **(B)**, guarantee period **(C)**, and the detections of spiked human serum samples **(D)**.

To verify the selectivity of the nano-biosensors for hnRNP A1 detection, immunoglobulin E (IgE), vascular endothelial growth factor (VEGF), human epidermal growth factor receptor 2 (HER2), human mucin 1 (MUC1), heterogeneous nuclear ribonucleoprotein A2 (hnRNP A2), and heterogeneous nuclear ribonucleoprotein B1 (hnRNP B1) were employed as interferents for comparison. The concentration of hnRNP A1 was fixed at 100 pg/mL, while the concentrations of these interferents were set at 10 ng/mL, which was 100 times of hnRNP A1 concentration. At the same time, the mixture was prepared with hnRNP A1 at 100 pg/mL and the interferents of 10 ng/mL. The current signals of these groups were presented in Figure 7A. It could be seen that the current signals of the mixed group and hnRNP A1 obtained a large increase even though hnRNP A1 concentration was lower. However, the interferents could not increase the current signals, which illustrated that the interferents could not combine with BC15. At the same time, the interferents could not interfere with the detection of hnRNP A1, demonstrating that the nano-biosensors possessed excellent selectivity.

Figure 7B showed the detection results under the identical conditions for 5 MG CEs, the relative standard deviation (RSD) for the detections was only 1.55% (p = 0.05), revealing small differences and dependable detection repeatability. The as-prepared FOαIT@Au/ssDNA/BC15/BSA nano-biosensors were stored in 4 °C refrigerator, and every 5 days, they were taken out to detect hnRNP A1 within 30 days. The current signal remained a minimum 98.17% of the result for the first detection (Figure 7C), which suggested a dependable long-term stability. For the detections of human serum samples, the human serum was obtainded from healthy people of Danyang People’s Hospital through centrifugal treatment of healthy blood, and the human serum was diluted for 20-fold by PBS buffer solution. In human serum, hnRNP A1 protein levels for healthy person was less than 10 ng/mL, while, that for patient increased to 20 ng/mL–100 ng/mL (Sun et al., 2024). In this work, the constructed nano-biosensors can detect the concentration of 0.1 pg/mL–10 ng/mL, suggesting that the nano-biosensors can detect 20 times concentration of 2 pg/mL– 200 ng/mL in human serum, which precisely met the detection range of its content in the serum. Figure 7D showed the detection results of the spiked hnRNP A1 in PBS and human serum under the same detection conditions. The results also revealed the obvious differences, and the detection results were listed in Table 2. The detection recovery was maintained at 104.57%–109.96%, all the recovery values were above 100%, the reason might be matrix effects of human serum samples; and the RSD was not more than 1.14% (p = 0.05), which certified the accuracy and credibility of the nano-biosensors for hnRNP A1.

**TABLE 2:** Detections of hnRNP A1 in spiked human serum samples with various concentrations (n = 3).

Sample	Spiked (ng/mL)	Measured value (ng/mL)	RSD (%)	Recovery (%)
1	0.1	0.1091	0.91	109.13
2	1.0	1.0996	0.87	109.96
3	10.0	10.4567	1.14	104.57

## 4 Conclusion

1. FOαIT MHNRs with an average diameter of 48 nm, an average length of 171 nm, and an Ms of 29.8 emu/g were successfully prepared at 400 °C for 2 h via a calcination-reduction process using β-FeOOH NRs, which were fabricated at 120 °C for 2 h via a hydrothermal process as precursors, and FOαIT@Au MNCs with AuNPs of 5 nm were obtained via the reduction of HAuCl_4_.
2. FOαIT@Au/ssDNA/BC15/BSA nano-biosensors were successfully constructed for hnRNP A1 detection. The biosensors exhibited a favorable linear relationship between the current and the logarithmic value of hnRNP A1 concentration (I (μA) = 3.897 l g C_hnRNP A1_ (pg/mL)+ 118.308, *R* ^2^ = 0.9990), a low LOD of 0.103 pg/mL, LOQ of 0.344 pg/mL, high sensitivity, excellent selectivity, satisfactory repeatability (RSD ≤1.55%), and dependable long-term stability, especially in the detection of spiked sample, the nano-biosensors demonstrated the dependable recovery (104.57%–109.96%, RSD ≤1.14%). All the results suggested a promising application prospect of the nano-biosensor in clinical examination, and once applied, the nano-biosensor would immensely promote the development of clinical tests.

## Data Availability

The original contributions presented in the study are included in the article/supplementary material, further inquiries can be directed to the corresponding authors.
